# Novel proteasome inhibitor delanzomib sensitizes cervical cancer cells to doxorubicin-induced apoptosis via stabilizing tumor suppressor proteins in the p53 pathway

**DOI:** 10.18632/oncotarget.23166

**Published:** 2017-12-12

**Authors:** Kevin Y. Guo, Lili Han, Xinyu Li, Andrew V. Yang, Jiaxiong Lu, Shan Guan, Hui Li, Yang Yu, Yanling Zhao, Jianhua Yang, Hong Zhang

**Affiliations:** ^1^ Department of Pathology, The University of Texas MD Anderson Cancer Center, Houston, TX 77030, USA; ^2^ Texas Children’s Cancer Center, Department of Pediatrics, Dan L. Duncan Cancer Center, Baylor College of Medicine, Houston, TX 77030, USA; ^3^ Department of Gynecology, People’s Hospital of Xinjiang Uyghur Autonomous Region, Urumqi, Xinjiang 830001, China; ^4^ Department of Pathology, Memorial Sloan Kettering Cancer Center, New York, NY 10021, USA

**Keywords:** cervical cancer, proteasome, delanzomib, doxorubicin, p53

## Abstract

Cervical cancer, the third most commonly occurring cancer, is the second leading cause of cancer related mortality among women. Aberrant ubiquitination and proteasome activity, both human papillomavirus and tumor derived, have been shown to contribute to tumor angiogenesis, proliferation, and invasion in many cancers, including cervical cancer. Thus, small molecule proteasome inhibitors are a potential and strategic treatment option for cervical cancer. In this study, novel proteasome inhibitor delanzomib (CEP-18770) exhibited potent pro-apoptotic and cytotoxic effects on a panel of cervical cancer cell lines by blocking proteasomal activity. Delanzomib also significantly sensitized cervical cancer cells to treatment of doxorubicin (Dox), a traditional chemotherapeutic agent. Furthermore, proteasome inhibition revealed stabilization of p53 and p53 transcriptional targets and induction of p38/JNK phosphorylation. Additionally, delanzomib worked synergistically with Dox to further upregulate p53 and its downstream targets and enhanced Dox-induced p38 phosphorylation. Our study strongly supports the 26S proteasome as a potential therapeutic target in cervical cancer and proteasome inhibition by delanzomib may be a potential treatment strategy for cervical cancer patients.

## INTRODUCTION

The third most common cancer worldwide, cervical cancer ranks second in cancer related mortality among women [1, 2]. Nearly 530,000 new cases emerge every year and cervical cancer accounts for over 275,000 deaths annually, making up 9% and 8% of total cancer rates, respectively [1]. Although incidence rates of invasive cervical cancer have decreased by 54% in the past 35 years, treatment still holds an unacceptably low 5-year survival rate of 75% for women younger than 65 [3]. Due to the derivation of cervical cancer from multiple human papillomavirus strains combined with viral cell cycle negative regulator inhibitors, researchers are encouraged to develop new methodologies and drugs to combat cervical cancer [4]. In parallel, a more complex biological understanding is necessary to implement novel therapeutic strategies to benefit patients with either HPV or cervical cancer.

The ubiquitin proteasome pathway plays a critical role in protein degradation and is widely conserved in eukaryotes. With the cascade mechanism of three variable enzymes, E1, E2, and E3, the 26S proteasome in humans is provided with a recognition signal to degrade proteins that have undergone specific ubiquitination [5]. Essentially, selective degradation via proteasome only occurs in proteins covalently ligated to multiple series of ubiquitin, or polyubiquitination [6]. Subunits Rpn13/ADRM1 and Rpn10/S5a and possibly Rpn1 and Rpt 5 of 26S proteasome bind polyubiquitinated proteins which pass through an unstructured initiation site, producing efficient proteasome-mediated degradation that is hastened by the natural occurrence of polyubiquitinated model substrates to facilitate gate opening and allosterically activate peptidase activities [7, 8]. In particular, proteasomal degradation regulates cell cycle protein regulators, such as but not limited to p27 and p53 [9]. Additionally, proteasome treatment offers possible pro- or anti-apoptotic effects induced via p38 and the c-Jun N-terminal kinase (JNK) pathway, notwithstanding the specific mechanisms are yet to be elucidated [10, 11]. As a positive side effect, lack of proteasomal degradation causes anti-tumor protein accumulation and induces a terminal unfolded protein response [12]. Studying proteasomal degradation is specifically fundamental to cervical cancer as there seems to be no connection with the normative mutations of tumor suppressive genes found in many other cancers, prompting heavy causation due to proteasome-related faults [13].

The wild type p53 gene codes for an invaluable transcription factor responsible for a variety of anti-proliferative programs in tumor cells [14]. In subsequent occurrence to DNA error, p53 protein accumulates, facilitating a transient arrest in the cell cycle, specifically either the G1 or G2 phase, allowing enzymatic repair of DNA lesions before DNA replication or mitotic division [15]. Such anti-tumor effects are accomplished via the protein activation of p21 for cell cycle arrest and Noxa to PUMA which induces mitochondrial cytochrome c induced apoptosis [16]. Another differential exists which p53 incurs apoptosis in cells with DNA damage [15]. In HPV positive cervical cancer cells which contain wild-type p53, initial levels of p53 are significantly downregulated since E6-AP, a member of the HECT family of E3 ligases, interacts with the E6 protein found in HPV, resulting in an E6/E6-AP complex that directly targets p53 for ubiquitination and eventual proteasomal degradation [17]. Thus, p53 is unable to be either stabilized or expressed in response to HPV E6 oncogene expression [18]. Likewise to E6-AP, MDM2 is an E3 ubiquitin ligase, promoting p53 degradation via the ubiquitin proteasome pathway [19].

Mitogen-activated protein kinases, or MAPKs, play a key role in regulation of a large variety of cellular behaviors predicated off of extracellular stimuli [20]. Two prominent subgroups in particular, the isoforms of the p38 MAPK and Jun N-terminal kinases, or JNKs, serve as regulators of apoptosis, autophagy, cell proliferation, cell migration, and invasion [20-22]. Upstream MAP kinase kinases or MKKs activate the dual phosphorylation sites located on p38 by isoforms, specifically MKK3 and MKK6 [23]. Also vulnerable to autophosphorylation, p38 MAPKs regulate a series of protein transcription factors including the tumor suppressor p53, activating transcription factors ATF-1/2/6, SRF accessory protein Sap1, CHOP derived from DNA inducible and growth arrest GADD153, ELK1, C/EBPβ, myocyte-specific enhancer factor 2 (MEF2), and high mobility group-box protein 1 (HBP1) [23-25]. Following activation, p38 proteins translocate into the nucleus and phosphorylate serine/threonine residues of related substrates [26]. Specifically, p38α MAPK activation has been shown to be crucial in chemotherapy induced apoptosis [27-29]. JNKs, another superfamily of MAPKs, play key roles in regulating cell proliferation, apoptosis, and cell differentiation [21]. Dual-specificity kinases MKK4 and MKK7 phosphorylate Thr- and Tyr- residues of JNKs upon TXF motifs within the respective activation loop [21]. Preceding MKK4 or MKK7 activation, fourteen distinct MAP kinase kinase kinases (MAP3Ks) serve to activate MAPKKs [30, 31]. All three JNKs, JNK-1, JNK-2, and JNK-3, prove significant roles in apoptotic signaling stimulation through activation of the cytochrome c-mediated death pathway [21]. Both p38 MAPKs and JNKs have been reported to be directly responsible for activation of p53 and p53 transcriptional targets [32, 33]. Consequently, lack of JNK function or inhibition of the JNK signaling axis promotes cervical cancer invasion [34]. Proteasome inhibition has proven useful in promoting JNK related apoptosis as proteasome inhibitors activate specific stress kinases effectively activating the JNK signaling pathway [11].

Tumor proteasome activity results in aberrant protein levels, accumulation of ubiquitin related enzymes, cell proliferation, and tumorigenesis [35, 36]. Indeed, small molecule inhibition of proteasome has been reported to have anti-tumor effects in a variety of human cancers, including multiple myeloma, acute myeloid leukemia, breast cancer, mantle cell lymphoma, non-small cell and small cell lung cancer (NSCLC and SCLC), melanoma, and pancreatic cancer [37-43]. Although several proteasome inhibitors have demonstrated anti-tumor effects in preclinical models or clinical trials, the novel proteasome inhibitor delanzomib (CEP-18770) has not been evaluated in patients with cervical cancer with or without high risk HPV [44-47]. Additionally, the unique combinations of delanzomib with traditional chemotherapeutic drugs have yet to be evaluated in cervical cancer within humans. Thus, the mechanisms of action of polychemotherapy have not been investigated.

Delanzomib, a novel 26S proteasome inhibitor, produces cytotoxic effects via inhibiting chymotrypsin-like activity of the proteasome with a significantly lower IC_50_ value than other proteasome inhibitors while having more favorable cytotoxicity regarding human epithelial cells, bone marrow progenitors, and bone marrow-derived stromal cells [48]. In this lab report, we show that by preventing 26S proteasome activity, delanzomib provided potent anti-tumor cytotoxicity at very low concentrations. As previously stated, low p53 and low activation of p38/JNK correlate with invasive cervical cancer and poor tumor prognosis. Delanzomib induced apoptosis by reversibly binding with the 26S proteasome and thus, blocked anti-tumor proteins from being degraded in cervical cancer cells. More importantly, in combination with doxorubicin, treatment was able to sensitize cervical cancer cells at very relatively low concentrations in comparison to respective IC_50_s and other lone chemotherapy drugs *in vivo*. The results of our experiment implicate that small molecule inhibitors such as delanzomib, both single and in combination with other chemotherapy treatments, should be developed for patients with cervical cancer and the 26S proteasome and the multiple associated pathways are potential therapeutic targets for cervical cancer.

## RESULTS

### Proteasome inhibitor delanzomib exhibits cytotoxic effects on cervical cancer cells

**Figure 1:**
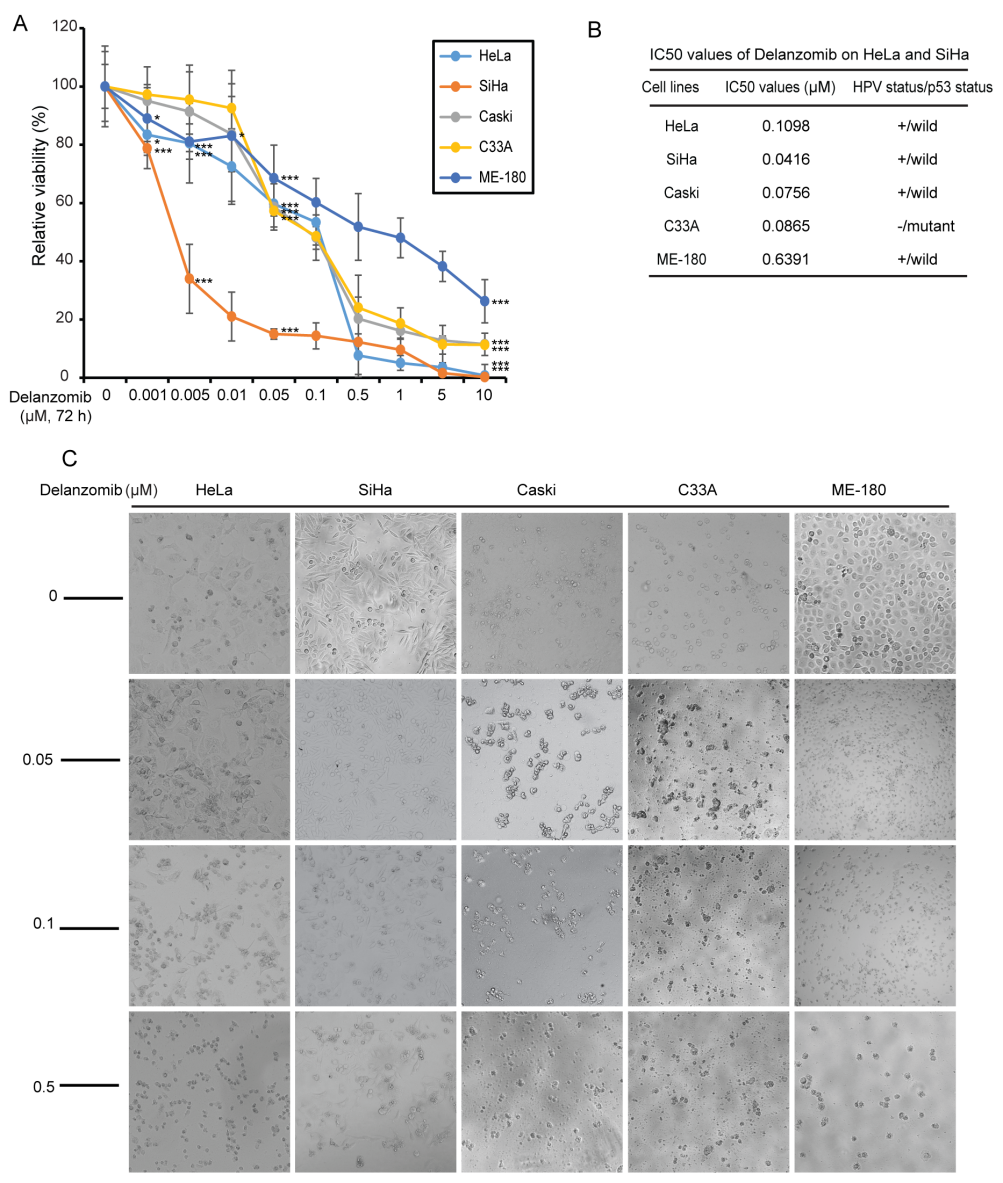
Delanzomib inhibits cervical cancer cell proliferation **(A)** Five typical cervical cancer cell lines were treated with increasing concentrations of delanzomib for 72 hours. Cell viability was then measured with Cell Counting Kit-8 (CCK-8) assay. *P*-values < 0.05 (^*^), *P* < 0.01 (^**^), or *P* < 0.001 (^***^) (Student’s *t*-test, two-tailed) were indicated. **(B)** The IC_50_ values of delanzomib in each cell lines listed were calculated based on the data in (A). Additionally, HPV/p53 status were shown for each cell line. **(C)** Morphologic changes of the five cervical cancer cell lines treated with increasing concentrations of delanzomib for 72 hours were shown.

To evaluate efficacy, delanzomib was tested on five typical cervical cancer cell lines: HeLa, SiHa, ME-180, C33A, and Caski cell lines. In a dose-dependent manner, delanzomib significantly reduced cell proliferation in the five cervical cancer cell lines tested (Figure 1A). Additionally, the IC_50_ values of delanzomib in all cell lines were relatively low (Figure 1B). Cell morphology imaging confirmed high apoptotic rates of all five cell lines in a dose-dependent manner (Figure 1C).

### Delanzomib induces apoptosis through upregulation of p53 and p53 transcriptional targets and p38/JNK phosphorylation in cervical cancer cells

**Figure 2:**
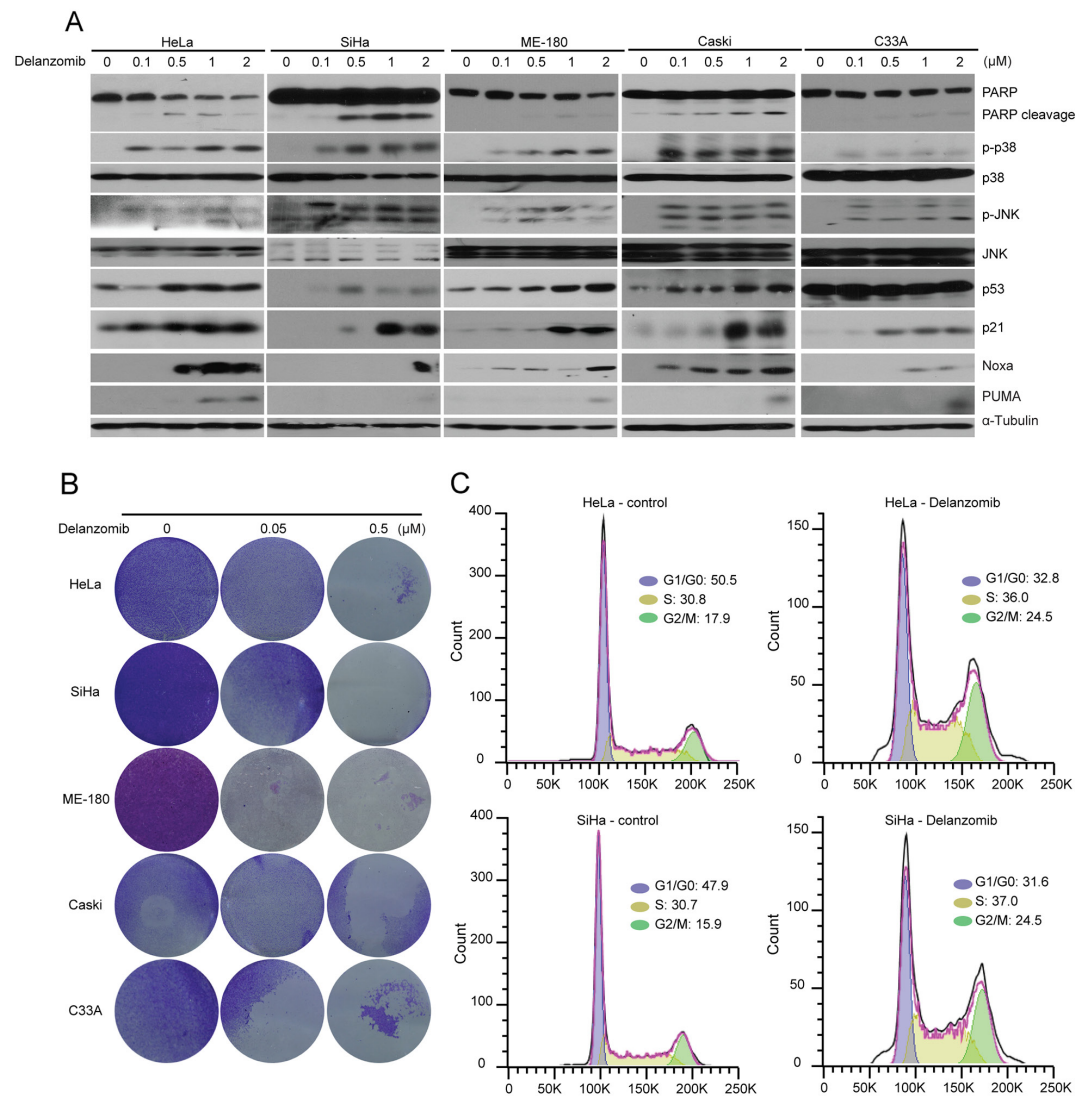
Delanzomib induces apoptosis through upregulation of p53 and p53 transcriptional targets and p38/JNK phosphorylation in cervical cancer cells **(A)** HeLa and SiHa cells were treated with indicated increasing doses of delanzomib for 24 hours, were subjected to SDS-PAGE, and then immunoblotted with indicated antibodies. Anti-α-Tubulin was used as a loading control for all whole cell extract samples. **(B)** A panel of five cervical cancer cell lines were treated with indicated doses of delanzomib (0 μM, 0.05 μM, 0.5 μM) and the crystal violet staining was shown. **(C)** HeLa and SiHa cells were treated with delanzomib (0.1 μM) for 12 hours. Cells were then washed in ice cold PBS, fixed with ice cold 70% ethanol, incubated in PI solution, and finally analyzed via flow cytometry. Cell cycle distributions of HeLa and SiHa were presented as percentages.

The p53 protein and its induction of downstream transcriptional targets, including p21, Noxa, and PUMA, are critical for tumor suppression and are invaluable for cancer treatment [14, 49]. To evaluate the mechanism behind the cytotoxic effect of delanzomib, we performed an immunoblotting analysis predicated upon p53, p21, Noxa, and PUMA. In a panel of 5 cervical cancer cell lines, a combination of initial protein levels of either p53, p21, PUMA, or Noxa were downregulated without treatment (Figure 2A, Supplementary Figure 1). In all cell lines tested, delanzomib was able to upregulate p53 levels and p53 downstream transcriptional targets p21, PUMA, and Noxa (Figure 2A). Both p38 and JNK are possible inducers of apoptosis and thus, we examined activation levels. We performed an immunoblotting analysis and found p38 and JNK activation in all cell lines with relatively low concentrations of delanzomib (Figure 2A). Additionally, delanzomib caused PARP cleavage, a confirmation of apoptosis (Figure 2A).

### Proteasome inhibitor delanzomib inhibits colony formation potential and induces G2/M phase arrest in cervical cancer cells

To test the ability of delanzomib to inhibit colony formation, we performed a colony formation assay. Delanzomib was able to inhibit colony formation with relatively low doses (Figure 2B).

In order to assess reasons behind apoptosis, we tested if delanzomib had potential to affect the cell cycle. Through cell cycle analysis, we observed significant accumulation of G2/M cell populations in cervical cancer cell lines HeLa (17.9% to 24.5%) and SiHa (15.9% to 24.5%) after 12 hours of treatment (Figure 2C).

### Delanzomib enhances the cytotoxic effect of Dox on cervical cancer cells

**Figure 3:**
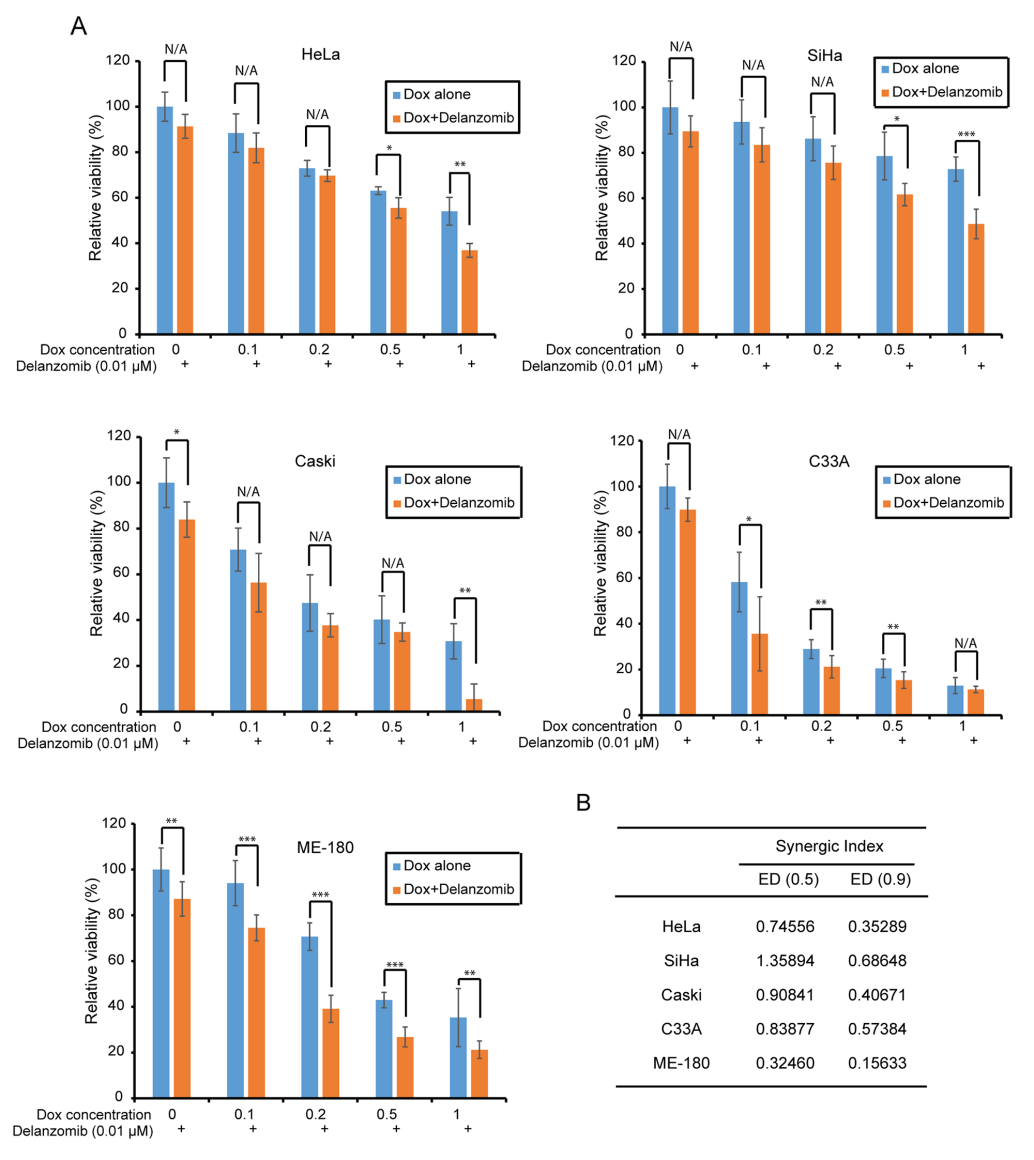
Delanzomib enhances the cytotoxic effect of Dox on cervical cancer cells **(A)** A panel of five cervical cancer cell lines were seeded in separate 96-well plates and were incubated with the indicated concentrations of Dox or Dox plus delanzomib (0.01 μM) for 24 hours. Cell viability was then measured with Cell Counting Kit-8 (CCK-8) assay. *P*-values < 0.05 (^*^), *P* < 0.01 (^**^), or *P* < 0.001 (^***^) (Student’s *t*-test, two-tailed) were indicated. **(B)** Synergic Indexes were shown with delanzomib and Dox.

Since cervical cancer cells are contingent upon proteasome dysregulation, traditional chemotherapeutic agents are unable to achieve a high enough efficacy and as a result, single treatment therapies will likely result in chemoresistance. Thus, a novel strategy such as combination therapy is required to overcome treatment resistance. To evaluate the effects of polychemotherapy, we tested delanzomib with the traditional chemotherapeutic agent doxorubicin (Dox) on a panel of five cervical cancer cell lines: HeLa, SiHa, ME-180, Caski, and C33A. We found that combination therapy with delanzomib and Dox significantly enhanced cytotoxic effects on cervical cancer cells (Figure 3A). At 50% and 10% cell viability, Synergic Indexes less than 1 had synergic interactions between delanzomib and doxorubicin (Figure 3B). Our data strongly suggest that delanzomib is able to increase Dox-induced cell death in all cervical cancer cell lines.

### Delanzomib enhances Dox-induced apoptosis through p53 stabilization, p21, PUMA, and Noxa upregulation, and p38/JNK activation in cervical cancer cells

**Figure 4:**
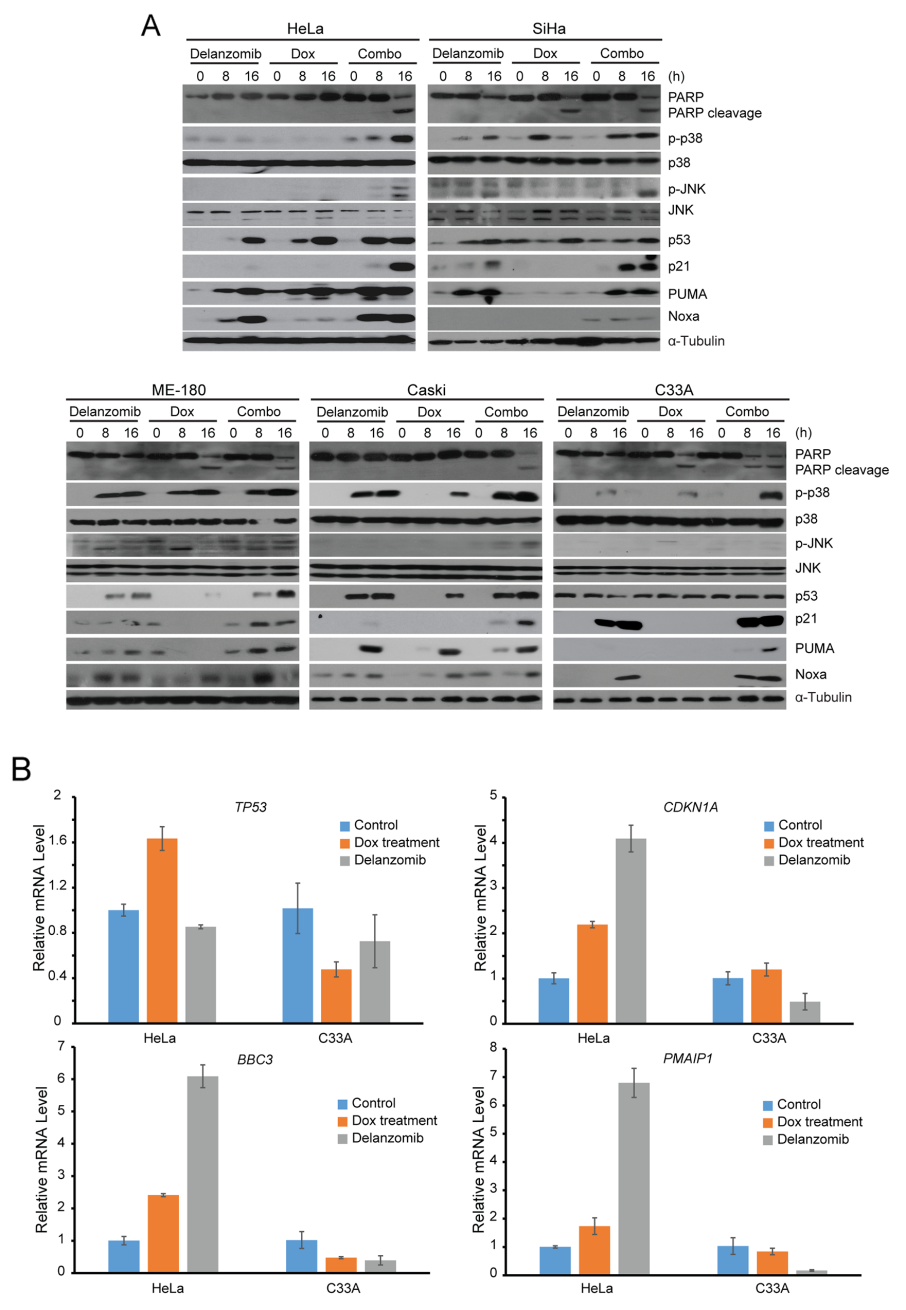
Delanzomib enhances Dox-induced apoptosis through p53 stabilization, p21, PUMA, and Noxa upregulation, and p38/JNK activation in cervical cancer cells **(A)** A panel of five cervical cancer cell lines were treated with either delanzomib (0.01 μM) alone, Dox (0.05 μM) alone, or delanzomib plus Dox for 0 hours, 8 hours, or 16 hours as indicated. Cells were subjected to SDS-PAGE and immunoblotted with indicated antibodies. Anti-α-Tubulin was used as a loading control for all whole cell extract samples. **(B)** mRNA levels of *TP53*, *CDKN1A*, *PMAIP1*, and *BBC3* are shown and indicated with either single or combination treatment. Cells were treated for 4 hours with either delanzomib (0.1μM) or doxorubicin (0.5 μM).

Since delanzomib works synergistically with Dox, we utilized an immunoblotting analysis to elucidate the mechanisms of action. On a panel of five cervical cancer cell lines, the combination of delanzomib plus Dox was able to accumulate levels of p53 where p53 was initially unnoticeable and upregulate p53 transcriptional targets p21, PUMA, and Noxa to a greater degree than just single treatment of delanzomib or Dox alone (Figure 4A). Since C33A is a p53 mutant cell line, initial levels of p53 were present without treatment (Figure 4A). However, both single treatment of delanzomib and combination treatment of delanzomib plus Dox caused upregulation of p53 transcriptional targets p21, PUMA, and Noxa in p53 mutant cell line C33A (Figure 4A). To assess whether p53 or its transcriptional targets were stabilized or activated, we performed RT-PCR to examine expressions of *TP53*, *CDKN1A*, *BBC3*, and *PMAIP1* for single treatments of delanzomib and Dox. RT-PCR revealed that in the wild-type p53 cell line HeLa, delanzomib treatment was able to stabilize p53 and activate p53 transcriptional target genes *CDKN1A*, *BBC3*, and *PMAIP1* (Figure 4B). Additionally, we found that treatment with Dox increased expression transcriptionally of p53, a possible explanation behind the synergy and greater levels of p53 due to delanzomib plus Dox treatment (Figure 4B). Since p53 was stabilized in HeLa, transcriptional targets of p21, PUMA, and Noxa were expressed to a greater degree (Figure 4B). However, in p53 mutant cell line C33A, *CDKN1A*, *BBC3*, and *PMAIP1* did not noticeably change in mRNA levels and thus, p53 downstream targets p21, PUMA, and Noxa were stabilized with treatment of delanzomib (Figure 4B). In addition, delanzomib increased Dox-induced activation of p38/JNK (Figure 4A), another possible explanation as to the greater cytotoxic effect. Furthermore, increased causation of PARP cleavage in delanzomib plus Dox treated cells confirmed greater cytotoxicity of the combination treatment (Figure 4A).

### Delanzomib inhibits colony formation of cervical cancer

**Figure 5:**
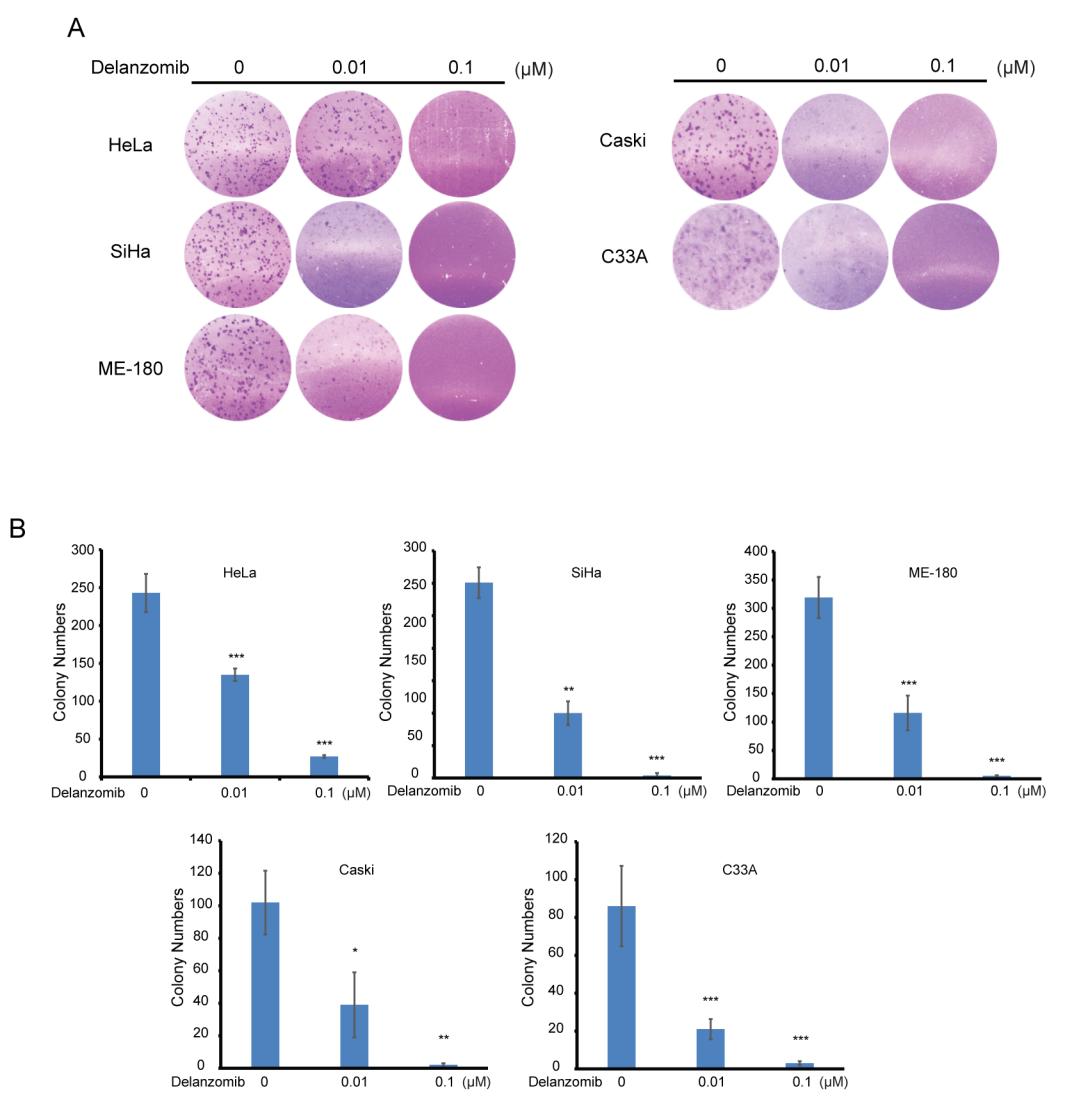
Delanzomib inhibits colony formation of cervical cancer **(A)** Five different cervical cancer cell lines were grown in soft agar, treated with indicated dosages of delanzomib, and grew in an incubated environment for 2 weeks. **(B)** Colony numbers of (A) were counted.

Aggressive tumor metastasis and neoplastic cells can often be characterized with anchorage-independent growth. Thus, we examined the effect of delanzomib on anchorage-independent cell growth of cervical cancer. On a panel of five cervical cancer cell lines, we found that delanzomib greatly inhibited colony formation in a dose-dependent manner (Figure 5A). Moreover, there was a statistically significant reduction in cell colony numbers as doses of delanzomib increased (Figure 5B), proving that delanzomib is capable of suppressing anchorage-independent growth of cervical cancer.

## DISCUSSION

The ubiquitin proteasome pathway is a potential target in cervical cancer due to the critical roles of the proteasome in many tumor related pathways. Thus, chemotherapeutic inhibition of the 26S proteasome is a viable strategy for cervical cancer treatment [50]. In this study, delanzomib induced apoptosis, sensitized cells to doxorubicin-induced apoptosis, and upregulated key proteins such as p53, p38, and JNK in multiple cervical cancer cell lines via proteasome inhibition.

The 26S proteasome, which affects a myriad of pathways and contributes to overall cell functionality, is an attractive target for cancer therapy [50]. As a result, potent proteasome inhibitor development is quickly becoming a novel chemotherapeutic hotspot. Bortezomib, the first generation of proteasome inhibitors, also inhibits proteasome function and induces apoptosis [51]. However, along with powerful proteasome inhibition, bortezomib induces sensory peripheral neuropathy, postural hypotension, and thrombocytopenia [44, 52]. Such adverse effects do little to ameliorate dosage limitations and results in a less desirable effect. On the other hand, delanzomib has been proven to act more favorably on human cells [48]. Although the mechanisms as to why delanzomib is more sympathetic to normal cells remain unknown, we hypothesize delanzomib might potentially be more applicable to treating patients with cervical cancer since reducing adverse side effects is a necessity when it comes to chemotherapy as both efficacy and side effects are strongly correlated due to dose limits.

One problem in the clinical setting is the inability of traditional chemotherapy drugs to kill all cells due to the chance of beneficial mutations in cancer cells that make treatment ineffective, a phenomenon known as chemoresistance. Relapse, a major effect of chemoresistance, remains a difficult problem to address. Although potent, doxorubicin, a traditional chemotherapeutic agent, lacks the ability to achieve a high enough efficacy as a stand-alone treatment. To make matters worse, Dox is heavily limited by its serious complications such as cardiomyopathy, which ultimately lead to lower doses and a less desirable effect [53]. Therefore, we tested whether delanzomib would work synergistically with Dox to exhibit a stronger cytotoxic effect. In terms of cell viability, combination treatment significantly increased apoptosis with concentrations lower than the control treatment of only doxorubicin. Treatment with doxorubicin plus delanzomib produced higher levels of PARP cleavage than single treatments alone, confirmation of stronger apoptosis. These results suggest that delanzomib is able to increase the efficacy of Dox by sensitizing cervical cancer cells and has the potential to lower doses of Dox and still achieve a significant antitumor effect.

Perhaps one of the most effectually complex proteins, p53 is a challenging target in cervical cancer due to the role of HPV E6 oncoprotein which directly causes specific ubiquitination and eventual degradation of p53 via E3 ubiquitin ligase E6-AP [54]. Thus, we hypothesized that preventing the proteasomal degradation will result in reclamation and stabilization of original p53 levels and possible upregulation of p53 transcriptional targets. We tested the effects of delanzomib on p53, p21, Noxa, and PUMA levels and found substantial upregulation of all respective proteins with relatively low concentrations of treatment. RT-PCR confirmed that the p53 was stabilized and indirectly activated genes coding for its downstream targets Noxa, PUMA, and p21. Our data strongly indicate that delanzomib stabilizes p53 by preventing degradation of ubiquitinated p53 and upregulates p53 downstream targets in wild-type p53 cells. Additionally, in wild-type p53 cells, doxorubicin has been proven to increase expression of p53 [55], which we concurrently observed in our results. The combination of increased p53 expression transcriptionally induced by Dox and stabilization of p53 by delanzomib is the underlying mechanism behind the synergy of the combination treatment. Although p53 levels did not significantly change in p53 mutant cell line C33A, treatment with delanzomib and delanzomib plus Dox caused stabilization of p53 transcriptional targets p21, PUMA, and Noxa. These results suggest that proteasome inhibition via delanzomib is a potential tumor suppressor and that the p53 pathway is one of the key mediators of proteasome inhibition-induced apoptosis. In addition, delanzomib was able to enhance Dox-induced p53 and p53 transcriptional target levels, an indication of synergy and greater cytotoxicity. Moreover, we also reasoned that delanzomib might have effects on p38 and JNK pathways since proteasome inhibition is a potential activator [22]. This is particularly important since both JNK and p38 are direct effectors of the intrinsic cell death pathway and are associated with p53 activation [32, 33, 56]. We showed that single compound treatment with delanzomib upregulates phosphorylation of both p38 and JNK.

In summary, this study demonstrates that delanzomib showed antitumor effects on cervical cancer by inducing apoptosis *in vitro* through p53, p38, and JNK pathways. Additionally, delanzomib increased Dox-induced cytotoxicity by upregulating p53 and p53 transcriptional targets in a panel of cervical cancer cell lines. Our data suggests that delanzomib is a promising cervical cancer treatment both as a monotherapy and in polychemotherapy.

## MATERIALS AND METHODS

### Antibodies and reagents

Proteasome inhibitor CEP-18770 was purchased from Selleckchem (S1157) (Selleckchem, Houston, TX, USA). Doxorubicin (Dox, D1515) was from Sigma (Sigma-Aldrich Corp, St. Louis, MO, USA). Anti-p21 (sc-53870), anti-α-Tubulin (sc-53646), anti-Ub (P4D1), and anti-p53 (sc-126) were from Santa Cruz Biotechnology (Santa Cruz Biotechnology, Dallas, TX, USA). Anti-PARP (9542L), anti-phospho-JNK (9251S), anti-JNK (9252L), anti-phospho-p38 (9211L), anti-p38 (8690S), anti-Noxa (14766S), and anti-PUMA (12450S) primary antibodies, as well as anti-Mouse (7076S), and anti-Rabbit (7074S) secondary antibodies were from Cell Signaling Technology (Cell Signaling Technology, Danvers, MA, USA).

### Cell lines and cell culture

Four human cervical cancer lines (SiHa, ME-180, C33A, and Caski) and one other human cervical cancer line (HeLa) used in the study were cultured in RPMI Medium 1640 (RPMI) and DMEM Medium (Lonza, Walkersville, MD, USA), respectively, with added 10% (v/v) heat-inactivated Fetal Bovine Serum (FBS) (SAFC Biosciences, Lenexa, KS, USA), 100 μg/mL streptomycin, and 100 units/mL penicillin. All cell cultures were maintained within a humidified incubator set at 5% CO_2_ and 37°C. All experiments were performed with cells under exponential growth conditions. The HeLa, SiHa, ME-180, C33A, and Caski cell lines were generously given from Dr. Ramondetta (UT MD Anderson Cancer Center, TX, USA).

### Cell viability assay

Assays utilized Cell Counting Kit-8 (CCK-8, WST-8[2-(2-methoxy-4-nitrophenyl)-3-(4- nitrophenyl)-5-(2,4-disulfophenyl)-2 H-tetrazolium, monosodium salt]) (Dojindo Laboratories, Rockville, MA, USA). 96-well clear-bottom plates were seeded with 1 x 10^4^ cells per well and after the media was changed 24 hours later, the plates were treated with various concentrations of CEP-18770, Dox, or their combinations. Subsequently, cells were maintained at 5% CO_2_ and 37°C for 24 hours if a combination treatment was administered or 72 hours if only a single treatment was administered. Old medium was then removed from each well and replaced with a mixture of 190 μL of RPMI Medium 1640 with 10% FBS and 10 μL of CCK-8; an hour later, via a microplate reader, absorbance was measured at 450 nm. Each experiment was performed in six replicates and background reading from the media was subtracted from each well. Synergic Index was calculated with ComboSyn Software (http://www.combosyn.com/).

### Cell imaging

All cervical cancer cells were cultured in 96-well clear-bottom plates at a start of 1 x 10^4^ cells per well. After 72 hours of treatment with indicated concentrations of CEP-18770, cell morphologies were photographed using an optical microscope. Each image was performed in triplicate and the representative was shown.

### Colony formation assay

Cells were seeded into 12-well plates at 2 × 10^3^ cells per well, then treated with delanzomib at indicated concentrations. After 72 hours, media containing delanzomib was replaced with fresh media. Two weeks later, cells were fixed and stained with 0.1% crystal violet and photographed. Each experiment was performed in a triplicate.

### Flow cytometry

Cells were treated with indicated concentrations of CEP-18770 and were subsequently washed with ice cold PBS. Next, cells were harvested and fixed with 70% ethanol. After centrifuging for 5 minutes at 500 g and 4°C, cells were stained with 50 μg/mL Propidium iodide (PI) solution and were analyzed by flow cytometry (FCS Express 4 Software). G1, S, G2/M cell cycle phases were shown.

### Immunoblotting

After treatment, harvested cells were washed with ice cold PBS twice and were lysed at 6°C in chilled RIPA buffer consisting of 50 mM Tris-HCl at pH 7.4, 150 mM NaCl, 1 mM EDTA, 1% NP-40, 0.25% sodium deoxycholate, 1 mM phenylmethylsulfonyl fluoride, 1 mM benzamidine, 10 μg/mL leupeptin, 1 mM dithiothreitol, 50 mM sodium fluoride, 0.1 mM sodium orthovanadate, and phosphatase inhibitor cocktail 2 and 3 (p5726 and p0044, Sigma). After centrifuging at 13,000 rpm for 15 minutes, supernatants were collected. To detect protein concentration, Bradford reagent (Bio-Rad Laboratories, Hercules, CA, USA) was used. Each sample was then mixed with loading buffer and was heated at 100°C. The protein solutions were separated by SDS-PAGE, transferred to polyvinylidene fluoride (PVDF) membranes (Bio-Rad), blocked with 5% milk for one hour at room temperature (25°C), and exposed to recommended dosages of indicated primary antibodies at 4°C overnight. Next, membranes were incubated with anti-mouse or anti-rabbit secondary antibodies conjugated with horseradish peroxide for one hour in room temperature (25°C). The ECL-Plus Western detection system (GE Health Care, Buckinghamshire, UK) was used to conduct chemiluminescent visualization. α-Tubulin was a loading control for whole cell extracts in all blots.

### Quantitative reverse transcription-PCR

Gene transcription levels were measured via the qRT-PCR method. Total RNA was extracted with TRIzol LS Reagent (Invitrogen) and the concentration of the RNA was measured subsequently after. Quantitative PCR was performed in a triplicate with SensiFAST SYBR Hi-ROX One-Step Kit in accordance to the manufacturer’s instructions (Bio-73005, Bioline). The mRNA levels for each gene was detected with Applied Biosystems™ Real-Time PCR Instruments. Primers used in this study were *TP53*: 5’-CAGCACATGACGGAGGTTGT, 3’-TCATCCAAATACTCCACACGC; *CDKN1A*: 5’-CTG AAGGGTCCCCAGGTG, 3’-CAGGCTT CCTGTG GGCGG; *BBC3*: 5’-TCAACGCACAGTACGAGCGG, 3’-AGGCACCTAATTGGGCTCC; *PMAIP1*: 5’-GGCTC CAGCAGAGCTGGAAG, 3’-GAAGGAGTCCCCTCA TGCAA.

### Soft agar

A 5% agar solution (214220, Difco Laboratories) was autoclaved and then kept in a 56°C water bath. A 2 mL 0.5% mixture of agar and either DMEM or RPMI 1640 medium containing 10% FBS was plated in 6-well plates as the function of a lower gel. A 1.5 mL 0.3% mixture of agar and either DMEM or RPMI 1640 medium containing 10% FBS was mixed with each cell line at a concentration of 1 x 10^4^ cells per well. The cells in soft agar grew at 37°C and 5% CO_2_ for 2 weeks and were stained with 0.005% crystal violet. Colonies were photographed and counted with VersaDoc Imaging System (Bio-rad). Each experiment was performed in a triplicate.

### Statistical analysis

For all *in vitro* assays, a two-tailed Student’s *t*-test was used to determine statistical significance. Values are presented as mean ± standard deviation (SD). P < 0.05 was considered to be standard for statistical significance in all assays.

### Synergic index calculation

Synergic Indexes were calculated using ComboSyn, Inc. software (https://www.combosyn.com). Synergic Indexes are shown in the format “Effect Dosage (ED) (percent cell viability).” ED (0.5) represents 50% cell viability and ED (0.9) represents 10% cell viability. Synergic Indexes lower than 1 represents an effective combination and the lower the number, the more effective the combination is.

## SUPPLEMENTARY MATERIALS FIGURE



## References

[1] Jemal A, Bray F, Center MM, Ferlay J, Ward E, Forman D (2011). Global cancer statistics. CA Cancer J Clin.

[2] Wright TC, Kuhn L (2012). Alternative approaches to cervical cancer screening for developing countries. Best Pract Res Clin Obstet Gynaecol.

[3] Brun JL, Stoven-Camou D, Trouette R, Lopez M, Chene G, Hocke C (2003). Survival and prognosis of women with invasive cervical cancer according to age. Gynecol Oncol.

[4] Crosbie EJ, Einstein MH, Franceschi S, Kitchener HC (2013). Human papillomavirus and cervical cancer. Lancet.

[5] Myung J, Kim KB, Crews CM (2001). The ubiquitin-proteasome pathway and proteasome inhibitors. Med Res Rev.

[6] Hershko A, Ciechanover A (1998). The ubiquitin system. Annu Rev Biochem.

[7] Bedford L, Paine S, Sheppard PW, Mayer RJ, Roelofs J (2010). Assembly, structure, and function of the 26S proteasome. Trends Cell Biol.

[8] Prakash S, Tian L, Ratliff KS, Lehotzky RE, Matouschek A (2004). An unstructured initiation site is required for efficient proteasome-mediated degradation. Nat Struct Mol Biol.

[9] Shen M, Schmitt S, Buac D, Dou QP (2013). Targeting the ubiquitin-proteasome system for cancer therapy. Expert Opin Ther Targets.

[10] Almond JB, Cohen GM (2002). The proteasome: a novel target for cancer chemotherapy. Leukemia.

[11] Meriin AB, Gabai VL, Yaglom J, Shifrin VI, Sherman MY (1998). Proteasome inhibitors activate stress kinases and induce Hsp72. Diverse effects on apoptosis. J Biol Chem.

[12] Obeng EA, Carlson LM, Gutman DM, Harrington WJ, Lee KP, Boise LH (2006). Proteasome inhibitors induce a terminal unfolded protein response in multiple myeloma cells. Blood.

[13] Ngan HY, Tsao SW, Liu SS, Stanley M (1997). Abnormal expression and mutation of p53 in cervical cancer--a study at protein, RNA and DNA levels. Genitourin Med.

[14] Zilfou JT, Lowe SW (2009). Tumor suppressive functions of p53. Cold Spring Harb Perspect Biol.

[15] Soussi T (2000). The p53 tumor suppressor gene: from molecular biology to clinical investigation. Ann N Y Acad Sci.

[16] Harris SL, Levine AJ (2005). The p53 pathway: positive and negative feedback loops. Oncogene.

[17] Beaudenon S, Huibregtse JM (2008). HPV E6, E6AP and cervical cancer. BMC Biochem.

[18] Hietanen S, Lain S, Krausz E, Blattner C, Lane DP (2000). Activation of p53 in cervical carcinoma cells by small molecules. Proc Natl Acad Sci U S A.

[19] Vu BT, Vassilev L (2011). Small-molecule inhibitors of the p53-MDM2 interaction. Curr Top Microbiol Immunol.

[20] Koul HK, Pal M, Koul S (2013). Role of p38 MAP kinase signal transduction in solid tumors. Genes Cancer.

[21] Dhanasekaran DN, Reddy EP (2008). JNK signaling in apoptosis. Oncogene.

[22] Choi CH, Lee BH, Ahn SG, Oh SH (2012). Proteasome inhibition-induced p38 MAPK/ERK signaling regulates autophagy and apoptosis through the dual phosphorylation of glycogen synthase kinase 3beta. Biochem Biophys Res Commun.

[23] Zarubin T, Han J (2005). Activation and signaling of the p38 MAP kinase pathway. Cell Res.

[24] Raingeaud J, Gupta S, Rogers JS, Dickens M, Han J, Ulevitch RJ, Davis RJ (1995). Pro-inflammatory cytokines and environmental stress cause p38 mitogen-activated protein kinase activation by dual phosphorylation on tyrosine and threonine. J Biol Chem.

[25] Wagner EF, Nebreda AR (2009). Signal integration by JNK and p38 MAPK pathways in cancer development. Nat Rev Cancer.

[26] Jin X, Mo Q, Zhang Y, Gao Y, Wu Y, Li J, Hao X, Ma D, Gao Q, Chen P (2016). The p38 MAPK inhibitor BIRB796 enhances the antitumor effects of VX680 in cervical cancer. Cancer Biol Ther.

[27] Furukawa T (2015). Impacts of activation of the mitogen-activated protein kinase pathway in pancreatic cancer. Front Oncol.

[28] Liu WH, Cheng YC, Chang LS (2009). ROS-mediated p38alpha MAPK activation and ERK inactivation responsible for upregulation of Fas and FasL and autocrine Fas-mediated cell death in Taiwan cobra phospholipase A(2)-treated U937 cells. J Cell Physiol.

[29] Ravindran J, Gupta N, Agrawal M, Bala Bhaskar AS, Lakshmana Rao PV (2011). Modulation of ROS/MAPK signaling pathways by okadaic acid leads to cell death via, mitochondrial mediated caspase-dependent mechanism. Apoptosis.

[30] Raman M, Chen W, Cobb MH (2007). Differential regulation and properties of MAPKs. Oncogene.

[31] Johnson GL, Nakamura K (2007). The c-jun kinase/stress-activated pathway: regulation, function and role in human disease. Biochim Biophys Acta.

[32] She QB, Chen N, Dong Z (2000). ERKs and p38 kinase phosphorylate p53 protein at serine 15 in response to UV radiation. J Biol Chem.

[33] Wei L, Zhu Z, Wang J, Liu J (2009). JNK and p38 mitogen-activated protein kinase pathways contribute to porcine circovirus type 2 infection. J Virol.

[34] Yuan B, Cui J, Wang W, Deng K (2016). Galpha12/13 signaling promotes cervical cancer invasion through the RhoA/ROCK-JNK signaling axis. Biochem Biophys Res Commun.

[35] Chen L, Madura K (2005). Increased proteasome activity, ubiquitin-conjugating enzymes, and eEF1A translation factor detected in breast cancer tissue. Cancer Res.

[36] Guo X, Wang X, Wang Z, Banerjee S, Yang J, Huang L, Dixon JE (2016). Site-specific proteasome phosphorylation controls cell proliferation and tumorigenesis. Nat Cell Biol.

[37] Moreau P, Richardson PG, Cavo M, Orlowski RZ, San Miguel JF, Palumbo A, Harousseau JL (2012). Proteasome inhibitors in multiple myeloma: 10 years later. Blood.

[38] Colado E, Alvarez-Fernandez S, Maiso P, Martin-Sanchez J, Vidriales MB, Garayoa M, Ocio EM, Montero JC, Pandiella A, San Miguel JF (2008). The effect of the proteasome inhibitor bortezomib on acute myeloid leukemia cells and drug resistance associated with the CD34+ immature phenotype. Haematologica.

[39] Agyin JK, Santhamma B, Nair HB, Roy SS, Tekmal RR (2009). BU-32: a novel proteasome inhibitor for breast cancer. Breast Cancer Res.

[40] Holkova B, Grant S (2012). Proteasome inhibitors in mantle cell lymphoma. Best Pract Res Clin Haematol.

[41] Scagliotti G (2006). Proteasome inhibitors in lung cancer. Crit Rev Oncol Hematol.

[42] Selimovic D, Porzig BB, El-Khattouti A, Badura HE, Ahmad M, Ghanjati F, Santourlidis S, Haikel Y, Hassan M (2013). Bortezomib/proteasome inhibitor triggers both apoptosis and autophagy-dependent pathways in melanoma cells. Cell Signal.

[43] Wente MN, Eibl G, Reber HA, Friess H, Buchler MW, Hines OJ (2005). The proteasome inhibitor MG132 induces apoptosis in human pancreatic cancer cells. Oncol Rep.

[44] Field-Smith A, Morgan GJ, Davies FE (2006). Bortezomib (Velcadetrade mark) in the treatment of multiple myeloma. Ther Clin Risk Manag.

[45] Potts BC, Albitar MX, Anderson KC, Baritaki S, Berkers C, Bonavida B, Chandra J, Chauhan D, Cusack JC, Fenical W, Ghobrial IM, Groll M, Jensen PR (2011). Marizomib, a proteasome inhibitor for all seasons: preclinical profile and a framework for clinical trials. Curr Cancer Drug Targets.

[46] Siegel DS (2013). From clinical trials to clinical practice: single-agent carfilzomib adverse events and their management in patients with relapsed and/or refractory multiple myeloma. Ther Adv Hematol.

[47] Garcia-Gomez A, Quwaider D, Canavese M, Ocio EM, Tian Z, Blanco JF, Berger AJ, Ortiz-de-Solorzano C, Hernandez-Iglesias T, Martens AC, Groen RW, Mateo-Urdiales J, Fraile S (2014). Preclinical activity of the oral proteasome inhibitor MLN9708 in Myeloma bone disease. Clin Cancer Res.

[48] Piva R, Ruggeri B, Williams M, Costa G, Tamagno I, Ferrero D, Giai V, Coscia M, Peola S, Massaia M, Pezzoni G, Allievi C, Pescalli N (2008). CEP-18770: a novel, orally active proteasome inhibitor with a tumor-selective pharmacologic profile competitive with bortezomib. Blood.

[49] Ryan KM, Phillips AC, Vousden KH (2001). Regulation and function of the p53 tumor suppressor protein. Curr Opin Cell Biol.

[50] Frankland-Searby S, Bhaumik SR (2012). The 26S proteasome complex: an attractive target for cancer therapy. Biochim Biophys Acta.

[51] Roccaro AM, Vacca A, Ribatti D (2006). Bortezomib in the treatment of cancer. Recent Pat Anticancer Drug Discov.

[52] Paramore A, Frantz S (2003). Bortezomib. Nat Rev Drug Discov.

[53] Chatterjee K, Zhang J, Honbo N, Karliner JS (2010). Doxorubicin cardiomyopathy. Cardiology.

[54] Kim YT, Zhao M (2005). Aberrant cell cycle regulation in cervical carcinoma. Yonsei Med J.

[55] Yeh PY, Chuang SE, Yeh KH, Song YC, Chang LL, Cheng AL (2004). Phosphorylation of p53 on Thr55 by ERK2 is necessary for doxorubicin-induced p53 activation and cell death. Oncogene.

[56] Cai B, Chang SH, Becker EB, Bonni A, Xia Z (2006). p38 MAP kinase mediates apoptosis through phosphorylation of BimEL at Ser-65. J Biol Chem.

