# Supplementary material for: Novel proteasome inhibitor delanzomib sensitizes cervical cancer cells to doxorubicin-induced apoptosis via stabilizing tumor suppressor proteins in the p53 pathway
Source: Oncotarget. 2017 Dec 12;8(69):114123–35. doi: 10.18632/oncotarget.23166 (PMC5768391; doi:10.18632/oncotarget.23166)
Supplement: Supplementary file 1 [file oncotarget-08-114123-s001.pdf]

Novel proteasome inhibitor delanzomib sensitizes cervical cancer cells to doxorubicin-induced apoptosis via stabilizing tumor suppressor proteins in the p53 pathway

SUPPLEMENTARY MATERIALS

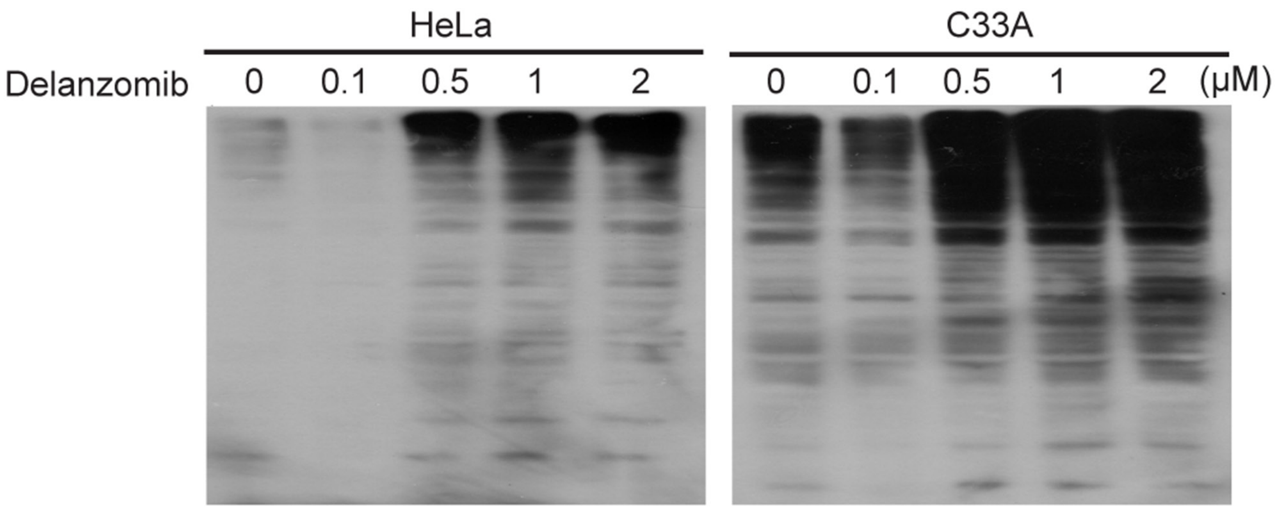

Supplementary Figure 1: Delanzomib increases ubiquitination of proteins. Immunoblot of ubiquitinated proteins were shown on delanzomib-treated HeLa and C33A in a dose-dependent manner.
